# Mycosinthetized Ag, CuO and ZnO nanoparticles from a promising *Trichoderma harzianum* strain and their antifungal potential against important phytopathogens

**DOI:** 10.1038/s41598-020-77294-6

**Published:** 2020-11-24

**Authors:** Verónica Fabiana Consolo, Andrés Torres-Nicolini, Vera Alejandra Alvarez

**Affiliations:** 1 Instituto de Investigaciones en Biodiversidad y Biotecnología (INBIOTEC-CONICET), Fundación para Investigaciones Biológicas Aplicadas (FIBA), Vieytes 3103, 7600 Mar del Plata, Argentina; 2 Instituto de Investigaciones en Ciencia y Tecnología de Materiales (INTEMA- CONICET-UNMDP), Av. Colón 10850, 7600 Mar del Plata, Argentina

**Keywords:** Biological techniques, Biotechnology, Microbiology

## Abstract

Fungal green biosynthesis of nanoparticles (NPs) is a promising eco-friendly method for mass-scale production. In the present study Ag, CuO and ZnO nanoparticles were biogenically synthetized using a cell filtrate of a strain of *Trichoderma harzianum* as a reducer and stabilizer agent. The structure, morphology and physicochemical properties of the NPs were characterized through transmission electron microscopy, dynamic light scattering, wide angle X-ray scattering and thermogravimetric analysis. Since nanotechnology could offer promising applications in agricultural area, we evaluated the ability of the NPs to reduce the growth of important fungal phytopathogens as *Alternaria alternata*, *Pyricularia oryzae* and *Sclerotinia sclerotiorum*. Silver and CuO NPs reduced significantly the mycelial growth of *A. alternata* and *P. oryzae* in a dose dependent manner. This is the first report of a multiple extracellular biosynthesis of NPs from *T. harzianum* and the first time that CuO and ZnO NPs were obtained from this fungus. In addition, we highlighted the rapid production of NPs, as well as, the potential of Ag and CuO for the control of phytopathogens. On the other hand, the three types of NPs could be easily and sustainably produced on a large scale with the chance of having multiple applications in biotechnological processes.

## Introduction

Nanoparticles (NPs) include microscopic material with a size dimension less than 100 nm, which have a great interest since they could be used in a number of processes that involves material science, agriculture, food industry, cosmetics, medical, and diagnostic applications^1^. Inorganic-based nanomaterials can be synthesized from pure metals as alumina, silver, gold, cadmium or from metal oxides such as CuSO_4,_ TiO_2_ and ZnO^2^. Among them, silver nanoparticles (AgNPs) are highlighted due to their antiviral and antimicrobial properties^3^. Some NPs like metal oxides, play a very important role in the production of sensors, surface coatings catalytic processes^4^, health area or antimicrobial, as well^5^.

Conventional physical and chemical methods for the synthesis of metal NPs, have high costs, give a low yield and require the use of toxic solvents which generate hazardous products for the environment^6^. Biological routes have been emerging as an economically feasible option to be produced in large volumes at the same size at reasonable costs because of their simplicity, and eco-friendly methods^7^. The absence of organic solvents and biosynthesis in aqueous phase under room pressure and temperature, are the main advantages^8^. Bacteria, algae, plants, diatoms and fungi are considered as NPs factories for their properties as reducing agents and stabilizers^9^. Fungi have special interest due to their rapid mycelial growth offering an increased surface area, easy handling of the biomass and culturing on a large scale. In addition, their ability to secrete significant amounts of proteins would amplify the nanoparticle synthesis productivity^10^.

The fungal genus *Trichoderma* (Ascomycetes, Hyprocreales) contains species that have a great economic importance owing to their production of industrial enzymes, antibiotics and bioactive metabolites^11^. Particularly, *Trichoderma harzianum* is the main mycoparasite used against plant pathogens. A literature review has shown that some *Trichoderma* species, including *T. harzianum*, can synthesize AgNPs, which have great potential as antimicrobials^12–14^. However, their ability in the synthesis of nanomaterials based on metal oxides, which could have multiple applications, has been little explored^15,16^.

In agriculture, the use of metallic NPs has a great potentiality to contribute in existing and future crop improvement^17^. They can be relevant in inhibiting plant pests, monitoring or detecting plant diseases and are a good alternative for dose reduction of chemical products as pesticides. Importantly, NPs could improve drug delivery or slow active ingredient release increasing their effectiveness^18^.

Silver nanoparticles present an antimicrobial activity against bacteria, fungi and viruses^3^. In agricultural production, the use of bulk copper compounds has been successful in reducing crop diseases caused by fungi and bacteria. They have an affordable cost and a low risk in generating pathogen resistance, due to their multi-site mode of action^19^. Particularly, copper NPs cause the alteration of protein expression as a key of inhibition of the microbial growth^20^. Moreover, ZnO NPs have been characterized to be effective on microorganisms, and mainly by their antibacterial properties based on their photo-oxidising and photocatalytic effect and regarded biosafety^21^.

Fungi are a major cause of about 70% of plant diseases^22^, producing substantial losses and causing a negative economic impact in several crops affecting the agricultural system worldwide^23,24^. Thus, some representative fungal species that massively infect different plant crops are: *Sclerotinia sclerotiorum,* affecting the production of soybean, tomato, lettuce, beans, and sunflower^25^, *Pyricularia oryzae* causing the most important disease in cereals as rice and wheat^26^ and *Alternaria alternata*, which is a saprobe, opportunistic and pathogenic fungus that has a wide host range, causing leaf spots and blights on many plant parts^27^.

In this context, although considerable work has been done testing the effects of NPs on plant pathogenic fungi, few of them were carried out with biogenically synthetized NPs^28^. Thus, the aim of this work attempted to evaluate the ability of a cell free extract from a native biocontroller strain of *T. harzianum* as a reducing agent and stabilizer to synthetize Ag, CuO and ZnO NPs. To our knowledge, few reports utilized *T. harzianum* only on the synthesis of AgNPs^13,14,29^, and there are no reports from this fungal specie on the synthesis of CuO and ZnO NPs from metal sulphate salts. Our results demonstrated the successful formation of three types metal NPs from a same *T. harzianum* strain using a simple green protocol. The biogenic NPs were characterized in order to confirm the synthesis and the structure, and to determine parameters including the size distribution, polydispersity index, zeta potential, and morphology. Moreover, their antifungal activity against phytopathogenous strains *A. alternata*, *P. oryzae* and *S. sclerotiorum* was demonstrated.

## Material and methods

### Fungal strain and biomass production

A *Trichoderma harzianum* strain from the INBIOTEC Culture Collection IB-363 was refreshed in 9 cm diameter Petri dish containing potato-dextrose agar (Britania) at 24 °C for 7 days. To generate fungal biomass for nanoparticle synthesis two agar plugs of young mycelia were harvested from the plates and transferred in 500 ml flasks containing liquid media broth as follow: KH_2_PO_4_ (7 g L^−1^); K_2_HPO_4_ (2 g L^−1^); MgSO_4_·7H_2_O (0.1 g L^−1^); (NH_2_)SO_4_ (1 g L^−1^); yeast extract (0.6 g L^−1^); glucose (10 g L^−1^). Flasks were incubated in an orbital shaker at 24 ± 2 °C and stirred at 150 rpm during 72 h in dark conditions. After growth, approximately 10 g of mycelia were harvested through a plastic sieve, washed with sterile double distilled water to remove broth medium components from the biomass. The fungal biomass was brought in contact with 150 mL^−1^ of sterile double distilled water for 48 h at 22 °C in 500 mL^−1^ flask. After incubation, the fungal biomass was separated from the aqueous cell free culture filtrate (CFCF) by passing it through Whatman filter paper no. 1.

### Synthesis of metal nanoparticles

Silver, copper oxide and zinc oxide nanoparticles were synthesized using 50 ml of aqueous CFCF in flasks by stirring their respective metal salt solution. For that, appropriate amount of AgNO_3_, CuSO_4_·5H_2_O and ZnSO_4_·7H_2_O was added in CFCF to make a final concentration of 1–2 mM solution. The reaction was carried out in dark conditions at 45 °C stirring vigorously. The effect of pH on the synthesis of nanoparticles was studied through experiments on a range of 6 to 12. The formation of nanoparticles was observed by their change of color and further validated using different techniques described below. Simultaneously, a positive control was the CFCF without silver nitrate, copper or zinc sulphate, and their respective negative control, 1–2 mM of each metal salt on deionized water, were also checked for comparison. The nanoparticles were separated by centrifugation, (8,000 rpm for 10 min) washed twice with double distilled water and dried by lyophilization during 24 h. After that, NPs were stored in polypropylene tubes in dark conditions and room temperature.

### Physicochemical characterization of metallic nanoparticles

A suspension of the synthesized Ag, CuO and ZnO NPs were diluted in ultrapure water, sonicated during 20 min at room temperature, and their size distribution was measured by dynamic fluctuations of light scattering intensity (DLS) using a Zetasizer Nano ZS, (Malvern Instruments Ltd., UK). The measurement gave the average hydrodynamic diameter of the particles, the peak values in the hydrodynamic diameter distribution, and the polydispersity index (PdI) that described the width of particle size distribution. The PdI scale ranges from 0 to 1 (with 0 being monodisperse and 1 being polydisperse)^30^. All measurements were carried out in triplicate with a temperature equilibration time of 1 min at 25 °C with an angle of 90 °C. The data processing mode was set to high multi-modal resolution. Thermogravimetric analysis (TGA) was carried on to determine NPs thermal degradation using a TGAQ500 V20.13 Build 39 (TA Instruments Co., USA) with approximately 10 mg of the samples, under N_2_ atmosphere and with a heating rate of 5 °C from room temperature to 900 °C under airflow.

Wide Angle X-ray Scattering (WAXS) measurement patterns of powdered Ag, CuO and ZnO NPs were recorded using XEUSS 2.0 XENOCS equipment. Patterns were registered with a 2D photon counting pixel X-ray detector Pilatus 100 k (DECTRIS, Swizerland) placed near the sample with a tilted angle of 36°. The scattering intensity, I(2θ), was recorded as a function of the scattering angle 2θ with λ = 0.15419 nm being the weighted average of X-ray wavelength of the *Cu-K*_*α12*_ emission lines. Due to the small beam size at the sample (1 mm × 1 mm) smearing effects were not taken into account. Samples were holded under vacuum within two kapton windows. Measurements were done in transmission mode. Each pattern was acquired at two different samples to detect distances for 10 min, in order to cover a 15° to 60° 2θ range.

Morphological visualization of nanoparticles by transmission electron microscopy (TEM) microscopy was carried out preparing a drop of aqueous Ag, CuO, or ZnO NPs samples included in a carbon-coated copper grid, dried and kept under vacuum before loading on a specimen holder. The images were obtained with a JEOL, JEM-2100 (Japan) instrument operating at an accelerating voltage of 200 kV with an energy dispersive spectrometer (EDS). Micrographs were obtained at 150,000 × magnification and the particles size distribution of NPs was evaluated using ImageJ 1.45 software.

### Biological activity of synthetized NPs

Effects of biogenically synthetized Ag, CuO and ZnO NPs on mycelium growth of plant fungal pathogens *A. alternata, P. oryzae* and *S. sclerotiorum* from the INBIOTEC Culture Collection were evaluated. Thus, a poisson assay was carried out placing a plug of 5 mm of young mycelium of each phytopathogen in the centre of a Petri plate containing potato-dextrose agar supplemented with 5, 10 and 20 ppm of each nanoparticle and kept for 7 days at 24 ± 2 °C under 12 h L/D photoperiod. Negative control cultures of fungal strains only grew in potato-dextrose agar. After that period, the diameter of the mycelium was measured. The growth effects of each type of synthetized NPs from the *T. harzianum* strain were already evaluated over their own growth. All the assays were performed in triplicate.

### Statistical analysis

In toxicological assays, mycelium development was compared with their respective negative control and the experimental data were statistically analyzed by one way analysis of variance (ANOVA). Means and standard deviations were calculated and examined by Tukey’s test at *p* < 0.05. The analysis was performed using GraphPad Prism v. 6.0 software.

## Results and discussion

### Biosynthesis and characterization of the nanoparticles

From aqueous CFCF of *T. harzianum,* silver, copper and zinc oxides NPs, were successfully synthetized in separate experiments after adding 1–2 mM silver nitrate, copper sulphate or zinc sulphate. Formation of metallic NPs was evidenced by changing the CFCF color suspension after their respective salt addition under continuous stirring at 45 °C in darkness conditions. Although pH reaction from 6 to 12 was evaluated, a rapid precipitation of NPs was observed within 10 min, when the initial CFCF at pH 6 was alkalinized with NaOH to pH 12. The reduction of Ag^+^ to Ag^0^ by the CFCF was immediately noticed after the addition of AgNO_3_ changing from pale yellow brown to colloidal dark brown due to the formation of AgNPs^30,31^. Meanwhile, CuO NPs formation occurred after reduction of sulphate ions from Cu^+2^ to Cu^0^, observed by an immediately change of the blue CFCF solution to a pale yellow brown was produced turning into a colloidal dark brown color after a continuous stirring^32^. In contrast, from colorless to a pale white color was observed in CFCF after the addition of ZnSO_4_, in which Zn^+2^ was reduced to Zn^0^ forming a solution of ZnO NPs evidenced by a fine white powder precipitation^33^, after the same conditions mentioned above. Hence, this is the first work in which a sole *T. harzianum* CFCF is able to synthetize three types of metallic NPs under the same conditions.

### Physicochemical properties of biogenic nanoparticles

**Figure 1:**
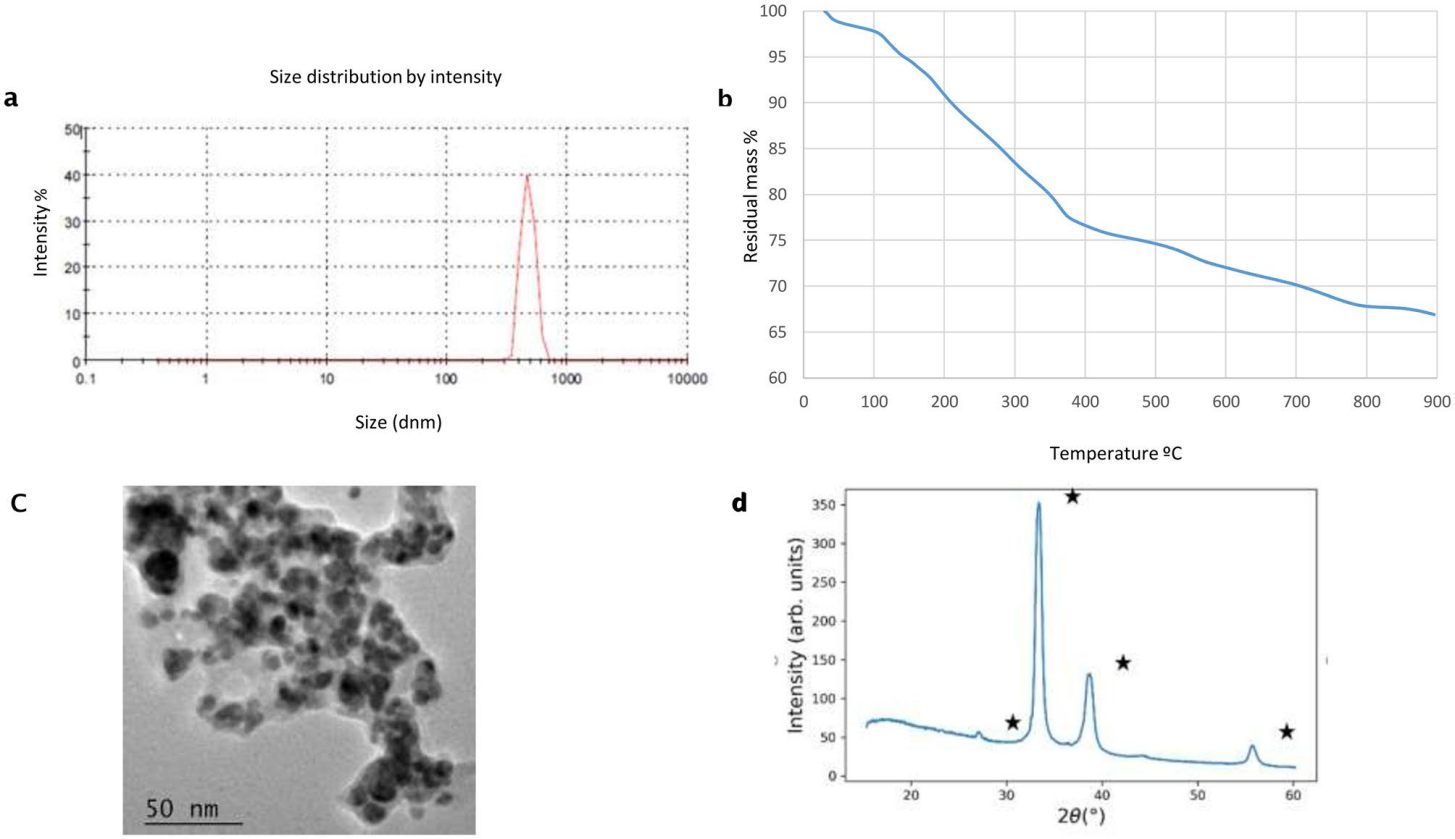
Size distribution and morphology of Ag nanoparticles biogenically synthesized with *Trichoderma harzianum* strain IB-363. (**a**) dynamic light scattering (DLS, n = 3), (**b**) nanoparticle tracking analysis (n = 3), and (**c**) transmission electron microscopy (TEM) coupled to energy dispersive X-ray spectroscopy (EDS) analysis (**d**) WAXS pattern, White stars represent Ag_2_O diffraction peaks (COD:00-101-0486).

**Figure 2:**
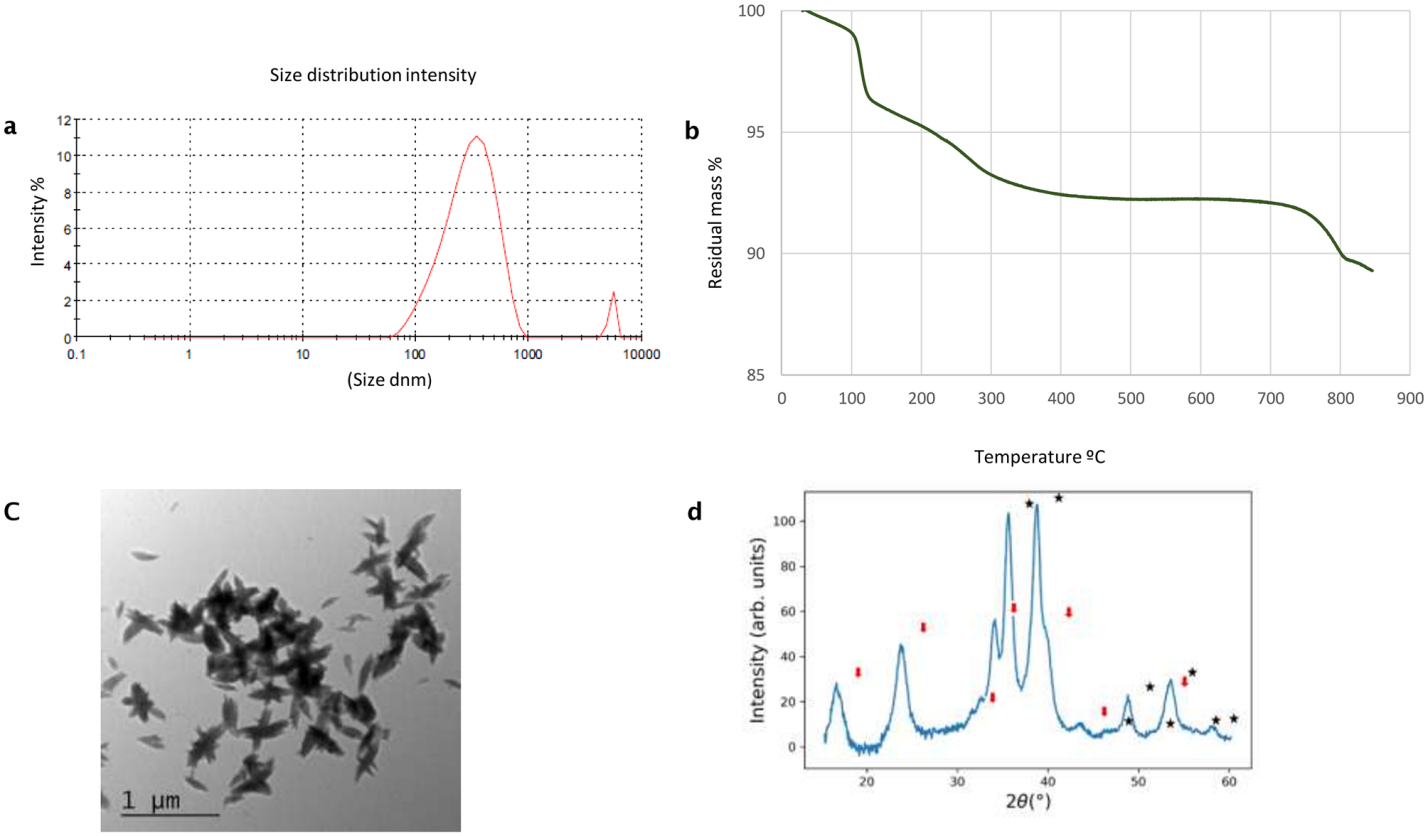
Size distribution of CuO nanoparticles biogenically synthesized with the fungus *Trichoderma harzianum* strain IB-363 (**a**) dynamic light scattering (DLS, n = 3), (**b**) nanoparticle tracking analysis (n = 3), and (**c**) transmission electron microscopy (TEM) coupled to energy dispersive X-ray spectroscopy (EDS) analysis (**d**) WAXS pattern for CuO NPs. Stars correspond to CuO diffraction 1 (00-721-2242) and red arrows to Cu (OH)_2_ (00-900-9157).

The size distribution of the biogenic AgNPs nanoparticles from *T. harzianum* determined by DLS showed an average of hydrodynamic diameter of 582.4 nm ± 112.78 nm with PDI value 0.36 ± 0.07 and Z average diameter 657.93 nm ± 127.62 nm. The distribution profile was monomodal (Fig. 1a) indicating low size variability and good physico-chemical stability. In the case of CuO NPs, two peaks were detected (Fig. 2a) ranging peak1 a size average of 276.43 nm ± 76.77 nm and peak 2, 58.87 nm ± 10.00 nm with a PDI value 0.49 ± 0.06 and Z average diameter 249.30 nm ± 61.24 nm. For ZnO NPs only one peak ranging in size 517.8 nm ± 50.1 nm was determined, and Z average diameter 792.4 nm ± 60.5 nm with a PDI value 0.563 ± 0.05 and a narrow distribution of ZnO NPs size was indicated in turns.

**Figure 3:**
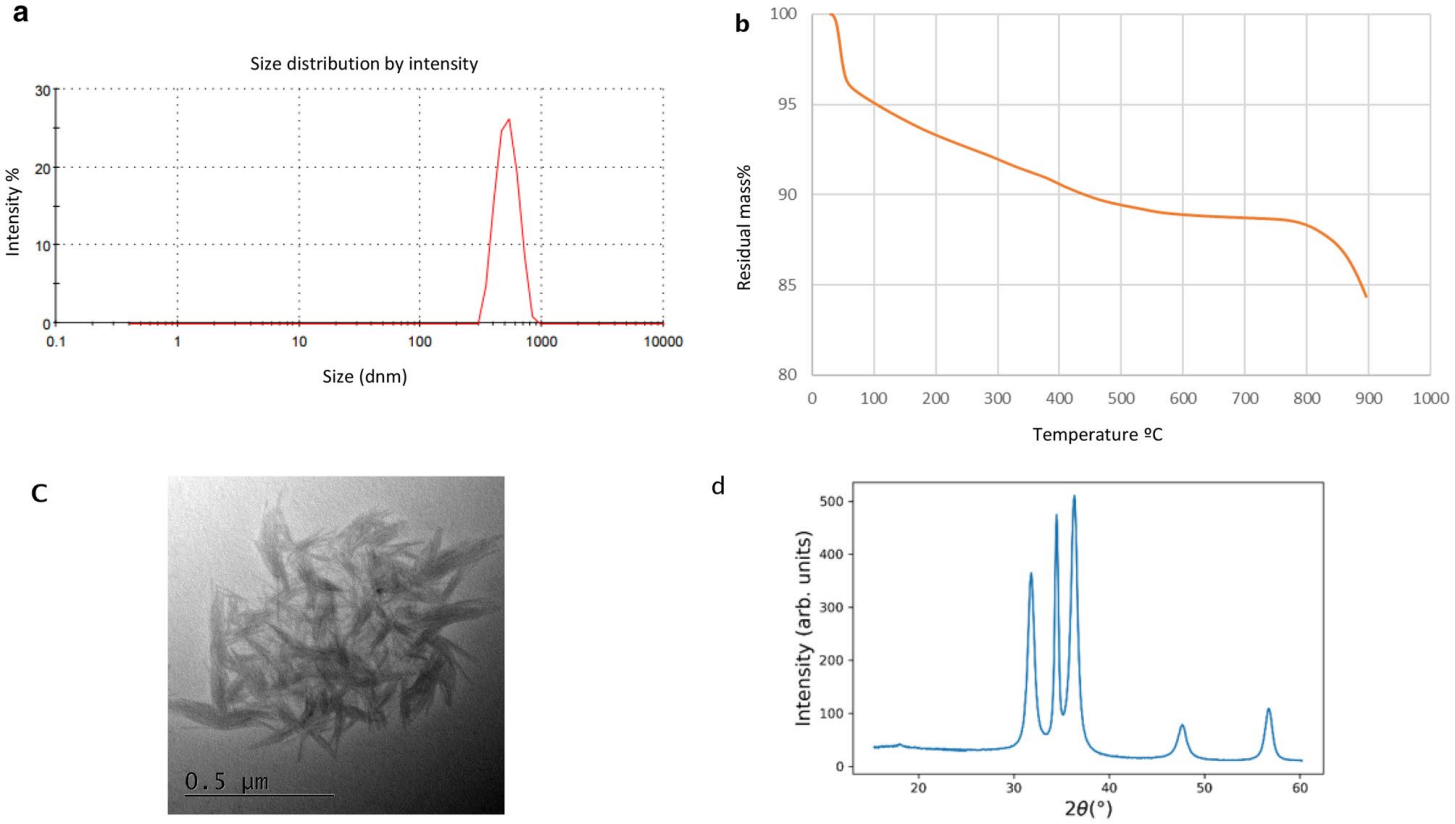
Size distribution of ZnO nanoparticles biogenically synthesized with the fungus *Trichoderma harzianum* strain IB-363. (**a**) dynamic light scattering [DLS, n = 3), (**b**) nanoparticle tracking analysis (n = 3), (**c**)] transmission electron microscopy (TEM) coupled to energy dispersive X-ray spectroscopy (EDS) analysis (**d**) WAXS pattern for ZnO NPs**.**

Thus, our results indicated the formation of three types of nanoparticles at the nanosize scale in an aqueous environment. It is remarkable that no weight gain occurred, as shown in Figs. 1a, 2a and 3a, suggesting that the biogenically synthesized Ag, CuO and ZnO NPs remained intact, with no oxidation under nitrogen atmosphere. In all of them PDI values suggest a low to a moderate polydispersity.

Thermal degradation analysis (TGA) of Ag, CuO and ZnO NPs from 25 to 900 °C under nitrogen atmosphere showed different aspects of each study NPs. For AgNPs and CuO NPs two major weight loss ranges were observed (Figs. 1b, 2b). In both, the first one centered at about 100 °C, in around 2% that can be related to the evaporation of adsorbed water. The second loss ranged between 100 and 400 °C, for AgNPs was about 25% and assigned to the thermal degradation of bioorganic compounds, maybe present on their surface^13,14,34^. For instance, CuO NPs weight loss was around 7%, which showed more than 90% thermal stability of the compound at 400 °C^35^. For both NPs the composition changes were completed at 400 °C, in which a steady weight loss was reached up to 900 °C. For instance, in the case of ZnO NPs a continuous high rate of mass loss has occurred between 300 and 900 °C in a range of around 15%, because of the evaporation of the water absorbed on the surface, and probably at higher temperatures due to the decomposition of zinc sulphate (Fig. 3b). This is the first report about biogenically synthetized CuO or ZnO NPs, through the TGA method using a CFCF from *Trichoderma* combined with sulphate salts. According to the literature, only for ZnO a similar degradation pattern, as observed in our study, is in concordance with Moharran et al.^36^ in which NPs were prepared using a physicochemical method based on hydrolysis and condensation of zinc acetate dihydrate by potassium hydroxide in alcoholic medium at low temperatures.

XRD analysis confirms the crystalline nature of NPs with peaks corresponding to Ag, Cu and Zn. The XRD pattern for AgNPs (Fig. 1d) revealed nanosilver at diffraction peaks at 38.11, 46.18, 63.44, 77.21 and 2.11 assigned. WAXS pattern shows coincident peaks with the calculated pattern of cubic Ag_2_O. As shown in Fig. 2d for CuO NPs, four strong absorbent peaks at angles of 29, 37, 44 and 62, respectively can be observed according to Khatami et al.^5^. The WAXS pattern, presents coincident peaks with the calculated pattern of monoclinic CuO, Additional peaks have been identified as Cu(OH)_2_, of orthorhombic crystalline structure. For ZnO NPs, as observed in Fig. 3d, the WAXS pattern shows that all peaks match with the calculated pattern of hexagonal ZnO. The differences in widths suggest the formation of anisotropic structures, with a preferential growth over the 002 direction.

Transmission electron microscopic images confirmed that three types of metal synthetized NPs are in nano-range. Shape of AgNPs was approximately spherical with little agglomeration aggregates, and a size distribution between 5 and 18 nm (Fig. 1c). Two possible explanations of this observation could be proposed: first, the agglomeration might appear due to a possible accumulation of proteins and enzymes, which were probably secreted during the biosynthesis, and second, the filtration might prevent the passing of different molecules of protein produced during the process of biosynthesis. The same type of the nanoparticles, obtained from our strain of *T. harzianum*, have a similar shape and size to the ones observed by Guilguer et al.^13^ and Elamawi et al.^30^ from *T. harzianum and T. longibrachiatum* respectively.

Dispersed and elongated fibres in shape for CuO NPs were observed (Fig. 2c) and sizes were between 38 and 77 nm in width and 135–320 nm in length. Thus, the morphology of biogenically CuO NPs synthetized in our study from *T. harzianum* is the first report up to date. In the literature the most commonly biogenic synthetized CuO NPs are mainly from different plant species in which spherical or cubic shapes were observed^37^. There are few reports of CuO NPs synthetized from *Trichoderma* species. The most related to our study is the intracellular synthesis from *T. koningiopsis* in which NPs were obtained from dead biomass where few aggregates showed a spherical shape with an average diameter of 87.5 nm^38^. More recently, CuO NPs were synthetized from CFCF of *T. asperellum* as dense agglomerate and spherical like shape^12^. Similar morphology of CuO NPs was observed by Zhu et al.^39^ whom prepared CuO NPs by adding NaOH solid into an aqueous solution of copper acetate after heating the solution from room temperature up to 100 °C. These conditions formed similar ellipsoidal particles of 100 nm in diameter as in our work. This procedure conditioned the nucleation and growth rate of CuO particles leading to the aggregation of CuO crystals and, yielding highly dispersed ellipsoidal particles. Moreover, Alishah et al.^40^, observed elongated CuO NPs obtained under hydrothermal synthesis conditions having an average diameter of 328.27 nm.

The morphology of biogenic ZnO NPs was like a fan and bouquet structure as shown in Fig. 3c. And sizes were between 27–40 nm in width and 134–200 nm in length. As proposed by Ludi and Niederberger^41^, this type of NPs structure was observed only under inorganic procedures of crystal formation using benzyl alcohol, in which primary nanocrystallites and their oriented attachment and surface reconstruction occur inside the agglomerates. Additionally, Pacholsky et al.^42^ reported that in alkaline water-containing solutions, the adsorption of OH or H_2_O as well counter ion, should be different. Thus, different surface charges could hinder or allow oriented attachment in different planes, as it occurs in crystal growth during biomineralization. It has been established, that pH takes an important role in modelling the morphology and the size of the NPs as well the pH increases, the number of nucleation centres, also increases^43^. Therefore, our study is the first report of an unusual ZnO NPs shape biogenically synthetized using the CFCF of *T. harzianum* determined by the alkaline environment.

As expected, the differences between sizes determined by DLS and TEM were due to characterization techniques. Although DLS shows the hydrodynamic radius of the nanoparticles, TEM shows their actual size. In consequence, DLS are bigger than real size of nanoparticles.

It is known that the influence of reaction conditions could have different effects in the morphology of synthetized nanocrystals. The conditions in our work were simple and affordable to fulfil, due to the temperature around 45 °C and the alkaline conditions after adding NaOH up to pH 12; all these favoured the rapid precipitation of three types of NPs.

### Potential of the biogenic NPs to control phytopathogenous fungi

**Figure 4:**
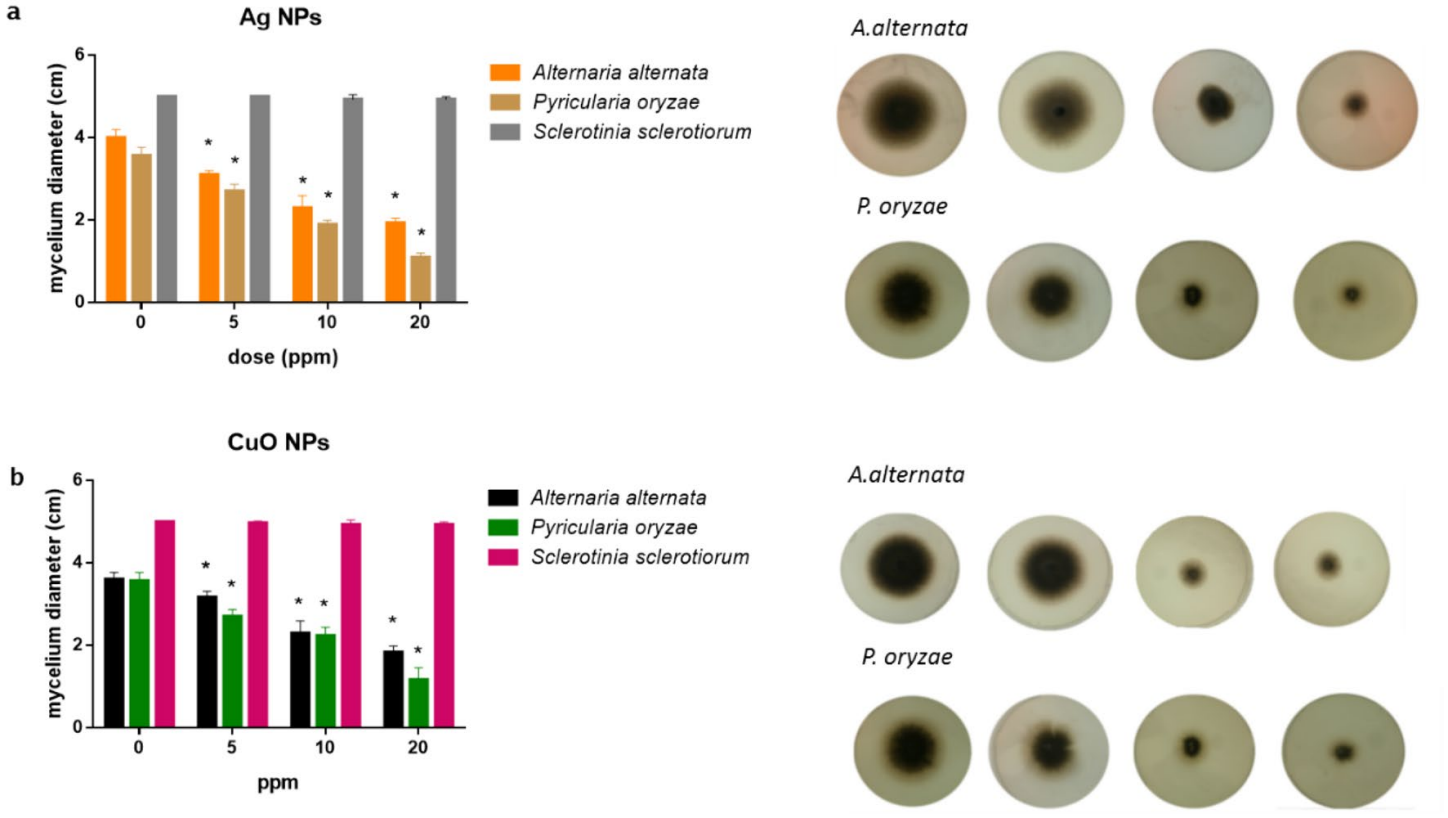
The inhibitory effect of mycelia growth on *Alternaria alternata* and *Pyricularia oryzae* on potato-dextrose agar medium containing (**a**) Ag NPs or (**b**) CuO NPs during 7 days.

The potentiality of Ag, CuO and ZnO NPs to control *A. alternata*, *P. oryzae* and *S. sclerotiorum* was assayed plating the fungal culture media supplemented with 5, 10 and 20 ppm of each type of NP and the diameter of the mycelium growth was measured after 7 days. Both Ag and CuO NPs caused a significant reduction in the mycelia development of *A. alternata* and *P. oryzae* in a dose dependent concentration. As shown in Fig. 4a under Ag treatment the mycelial diameter for *A. alternata* was reduced 18% at 5 ppm, 42% at 10 ppm and 52% at 20 ppm. In other hand, the mycelial growth of *P. oryzae* was reduced 22%, 46% and 68% for each respective NPs doses. Moreover, under CuO treatment a similar inhibitory effect than Ag was observed (Fig. 4b). Both under Ag and CuO treatment, although no effect in the diameter of mycelia was observed for *S. sclerotiorum,* a slightly mycelia and a minor size of sclerotia with a distribution on the edge of the plate, was observed (not shown). On the other hand, under treatment with ZnO NPs, despite there was a tendency to reduce mycelial growth of plant pathogens, these results were not statistically significant (data not shown).

Interestingly, the three biogenically synthetized NPs have not shown deleterious effects over *T. harzianum* development in all the concentrations tested, in comparison with their negative control. As reported fungal species as *T. harzianum* and *T. asperellum* are able to metabolize different heavy metals^44^, therefore, it is not surprising that if *T. harzianum* could be applied combined with nanoparticles, but the development would not be affected.

Although effects of AgNPs toxicity was demonstrated against different fungal plant pathogens^14^, this is the first time that *A. alternata*, *P. oryzae* and *S. sclerotiorum* were assayed simultaneously with three types of NPs synthesized from a single *T. harzianum* strain. We propose, that given the same toxicity effect between silver and copper NPs over these phytopathogens, maybe CuO NPs could be more advantageous for their use in the agriculture environment since they are less toxic than AgNPs.

Since ZnO NPs were not significantly toxic to the fungal pathogens assayed in this study, other microorganisms could be considered as a target for further assays. Moreover, these types of NPs have been reported as very effective antibacterial agents against a broad spectrum of bacterial species, and because of their high surface in comparison with their volume ratio gives them unique physicochemical properties^45^. In addition, other studies have demonstrated the fungistatic potential of ZnO NPs against other fungal pathogens including *Fusarium* sp., *Botrytis cinerea*, *Penicillium expansum*, *Aspergillus niger* and *Rhizopus stolonifer*^18,46,47^.

In later studies, maybe another possibility to enhance ZnO effect against phytopathogens could be a possible fusion of NPs to create bi-metallic nanoparticles as demonstrated by Paszkiewicz et al.^48^ in which study they found that the fusion of Cu and Ag increased the strength of their toxicity as antibacterial and antifungal compound.

## Conclusions

In this work, the successful synthesis of three types of NPs Ag, CuO and ZnO from *T. harzianum* was carried out following a green, affordable and non-toxic method. This is the first report in which the aqueous cell free culture filtrate (CFCF) from a sole strain of the fungus, is able to synthetize different types of metal NPs using a one step protocol based on alkaline conditions. These promising results provide new insights to produce different NPs taking advantage of the simplicity of the method maximizing the potential of a single strain.

Physicochemical properties of each metallic NPs showed a narrow size distribution, thermal stability and crystalline XRD spectra, and remarkably up to date, the morphology observed for CuO and ZnO NPs is rare. The antimicrobial efficiency of Ag and CuO NPs presented similar inhibitory effects against the radial growth of the mycelia of *A. alternata, P. oryzae* and sclerotia formation of *S. sclerotiorum* in a dose dependent manner, highlighting their potentiality for agricultural applications. In spite of, not having antifungal properties ZnO NPs can be assayed against other target microorganisms. Remarkably, NPs obtained in this study could have a strong application in other areas as pharmaceutical, cosmetics, clothing and food industry, as well. In conclusion, the use of the non-pathogenic fungus *T. harzianum* could be a good tool to handle the production of in a large scale.
